# Pediatric trauma volume fell during the initial COVID-19 wave but rebounded to new highs for the remainder of 2020

**DOI:** 10.5249/jivr.v16i1.1771

**Published:** 2024-01

**Authors:** Zachary T. Sheff, Brett W. Engbrecht

**Affiliations:** ^*a*^ Peyton Manning Children’s Hospital, 2001 W. 86th Street, Indianapolis, IN 46260, United States.

**Keywords:** Pediatrics, Trauma, Injury, Epidemiology, COVID-19

## Abstract

**Background:**

Previous studies have shown a reduction in pediatric trauma volume during COVID-19, but many have looked at a limited number of facilities, analyzed a narrow timeframe, or both. The objec-tive of this analysis was to assess the impact of COVID-19 on pediatric trauma volume for a statewide sample during 2020. Based on previous literature, researchers hypothesized a reduction in volume during the implementation of these policies.

**Methods:**

Retrospective cross-sectional analysis of five years (2016 – 2020) of Indiana’s statewide trauma patient registry. Patients under age 15 were included. Those who were transfer patients or missing key data were excluded. In total, 10,926 patients were included in analysis. Baseline years (2016 – 2019) were compared to 2020 to estimate the impact of COVID-19 on pediatric trauma volume.

**Results:**

Overall monthly volume of pediatric traumas were lower than baseline in March and April 2020 (though not significantly), but rebounded quickly and were above trend in the latter half of the year. Injury patterns differed in both mechanism and location from previous years. Gunshot wounds were more prevalent than previous years, while the volume of non-accidental traumas fell slightly. Injuries that occurred in private residences rose significantly, while fewer took place in schools.

**Conclusions:**

Results indicated an initial drop in injury volume consistent with previous findings, but these were offset by increased volume in the second half of 2020. The growth in gun violence is concerning and warrants additional research. Changes in behavior in response to the pandemic such as re-duced participation in sports and use of playgrounds, reduced driving, and increased time at home help explain the changes observed in injury patterns. These findings emphasize the continued need for pediatric trauma care during the pandemic.

## Introduction

The COVID-19 pandemic caused massive disruption to health systems around the world, including trauma services. Health systems responded to the initial outbreak by reducing or eliminating elective procedures, preserving intensive care unit (ICU) capacity, and asking the public to forgo trips to the emergency department (ED) in all but the most extreme circumstances.^1^ Demand for hospital services was further reduced during the initial stages of the COVID-19 outbreak by policies such as stay-at-home (SAH) orders and cancellation of in-person school and childcare services. 

Studies conducted on this time period have repeatedly shown a reduction in overall trauma volume.^2-5^ Similar reductions in pediatric trauma volume have also been found. ^6-10^ These findings were not limited to a specific geographic region and reflected reduced volume across many types of trauma. Notable exceptions included violent and non-accidental trauma (NAT) which remained constant—or increased—during the COVID era.^10-12^ Many investigations of the impact of COVID-19 on pediatric trauma volume have focused on a narrow time period in the spring of 2020, used data from a small number of facilities, or both. In fact, only one recent study has looked at a large, multicenter sample—and also concluded that overall pediatric trauma volume fell during 2020.^10^

Contrary to this prevailing trend in this literature, our analysis finds that pediatric injury volume increased by 14 percent compared to previous years. We use Indiana’s pediatric trauma registry to produce a large, multiyear sample of pediatric trauma cases for an entire state. These data provide a comprehensive picture of pediatric trauma injuries before and during the first waves of the COVID-19 pandemic in Indiana. 

Using the statewide registry, the objective of this analysis was to assess the impact of COVID-19 on pediatric injury patterns in Indiana by comparing 2020 volumes to the previous four years. Based on findings from previous studies, researchers hypothesized that overall volume would decrease in 2020. In addition to overall pediatric trauma volume, secondary analyses investigated specific mechanisms of injury (gunshot wounds (GSWs) and NAT) and locations at which injuries occurred (private residences and schools).

## Methods 

This is a retrospective cross-sectional analysis of Indiana’s statewide trauma patient registry. The study protocol was approved by the institution’s Internal Review Board prior to data collection and analysis. The data used in this analysis were obtained through a data request to the Division of Trauma and Injury Prevention at the Indiana Department of Health which maintains the Indiana Trauma Registry. This registry contains patient data from over 100 hospitals in Indiana representing nearly every trauma case that occurs in the state. Data were collected in accordance with the National Trauma Data Standards put forth by the American College of Surgeons (ACS) with inclusion criteria generalizable across the US.^13^

**Figure 1:**
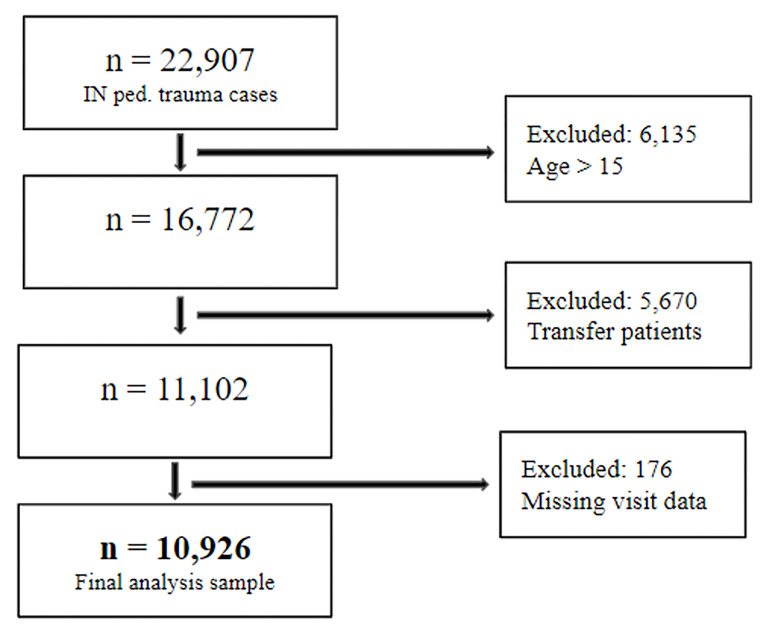
Inclusion and exclusion diagram.

Data were requested for all patients age 18 or under who were included in the trauma registry for calendar years 2016 through 2020. Final inclusion criteria for this analysis limited patients to those under age 15 (to better align with ACS definitions of pediatric trauma), excluded patients who were transfers, and excluded patients who were missing key variables (Figure 1). Ultimately, 10,926 patients were included in the analysis.

After applying inclusion criteria, data were aggregated into monthly observations that included measures of volume by treatment type (ED, hospital admission, ICU or ventilator utilization), by injury type (falls, motor vehicle collisions (MVCs), pedestrians struck, and GSWs, individuals struck by objects, and NAT), and by injury location (private residences, schools, outdoors/sports, and streets/highways). Additionally, demographic variables and measures of injury severity were included in the data set. 

For analysis, calendar year 2020 was compared to baseline years (2016 – 2019) on a monthly basis. Outcome measures for each month in 2020 were compared to their counterparts in 2016 – 2019 to estimate the divergence from baseline trends (e.g. January 2020 compared to the average of January 2016, January 2017, January 2018, and January 2019). This allowed us to track the evolution of the changing trend in pediatric trauma volume throughout 2020.

This structure allowed for comparisons between 2020 and baseline years at different stages of the pandemic and in relation to policy responses to the pandemic. Key policies include Indiana’s SAH order which ran statewide from 3/25/20 through 5/18/20 and the switch to remote learning for all K-12 public schools (3/19/20 through 7/31/20). Indiana’s most populous county, Marion County, maintained its SAH order slightly longer than the rest of the state and eased its initial SAH order on 5/31/20.

Descriptive tables were constructed that compare historic data (2016 – 2019) to 2020. Comparisons in these tables were assessed for statistical significance using Wilcoxon rank-sum tests and chi-square tests as appropriate. Outcomes in the months of 2020 were compared to baseline values using a linear fixed-effects regression framework in which the monthly outcome of interest was included as a dependent variable and regressed against a suite of dummy variables that indicated the month of the year to which the observation belonged. This structure allowed coefficients to be tested for statistical significance. Comparing the same months across years accounted for seasonal trends in pediatric trauma volume.

## Results

Descriptive statistics comparing pediatric trauma injuries in Indiana between baseline years (2016-2019) and 2020 are shown in Table 1. Overall volume was 14 percent greater in 2020 than in baseline years (2016 – 2019 average annual volume = 2,125; 2020 volume = 2,425). The proportion of injuries that included an ED visit was greater in 2020 than baseline years (97.8% vs. 96.6%, respectively), but the proportion of injuries admitted to a hospital (71.1% vs. 79.5%) or admitted to an ICU (13.5% vs. 15.7%) were lower in 2020. Patient demographics (age, sex, race, and ethnicity) did not differ from previous years. The mortality rate was comparable to previous years, though injury severity was slightly lower in 2020 (2016 – 2019 median injury severity score (ISS) = 5, 2020 median ISS = 4). 

Mechanism of injury was distributed differently in 2020 than baseline years (p <0.001, Table 1. The most common mechanism of injury, falls, accounted for a smaller proportion of injuries in 2020 than baseline years (42.0% vs. 46.1%, respectively). Other mechanisms varied slightly from previous years; however, GSWs increased notably in 2020, representing over a 50 percent increase from baseline years (52 GSWs in 2020 accounting for 2.2% of all injuries vs. 32 annual GSWs in baseline years accounting for 1.5% of all injuries).

**Table 1:** Descriptive statistics comparing 2016-2019 to 2020.

		2016-2019	2020	p-value
	Total volume	2,125	2,425	
	ED visits	2,054 (96.6)	2,371 (97.8)	0.005
	Hospital admits	1,690 (79.5)	1,724 (71.1)	<0.001
	Admits w/ ICU	334 (15.7)	328 (13.5)	0.010
Demographics				
	Age	6 [3-10]	6 [3-11]	0.586
	Female	811 (38.2)	966 (39.8)	0.144
	White	1,704 (80.2)	1,941 (80.0)	0.914
	Hispanic	129 (6.0)	163 (6.7)	0.242
Injury severity				
	Mortality	35 (1.6)	36 (1.5)	0.697
	ISS	5 [4-9]	4 [4-9]	0.003
Mechanisms				
	Fall	981 (46.1)	1,018 (42.0)	<0.001
	MVC	304 (14.3)	312 (12.9)	
	Pedestrian struck	209 (9.8)	289 (11.9)	
	GSW	32 (1.5)	53 (2.2)	
	Struck by object	197 (9.2)	195 (8.0)	
	NAT	173 (8.1)	162 (6.7)	
	Other mechanism	330 (15.5)	478 (19.7)	
Location of injury				
	Home	786 (37.0)	1,244 (51.3)	<0.001
	School	92 (4.3)	64 (2.6)	
	Outdoors/sports	286 (13.5)	287 (11.8)	
	Street/highway	316 (14.8)	301 (12.4)	
	Other location	647 (30.4)	529 (21.8)	

Numbers in parentheses indicate percentages. Numbers in square brackets indicate interquartile range. Age and injury severity score (ISS) are given as medians and all other variables are described by counts and percentages. Counts for baseline years are annualized for ease of comparison with 2020 numbers. P-values are from Wilcoxon sum-rank tests for age and ISS and chi-square tests for all other measures. P-values less than 0.05 were considered statistically significant.

The location at which injuries took place also differed in 2020 compared to previous years (<0.001, Table 1). Over half of injuries took place in private residences (51.6%) in 2020, up from an annual average of 37 percent from 2016 to 2020. On the other hand, fewer injuries took place at schools during 2020 than baseline years (2.6% vs. 4.3%, respectively). 

**Figure 2:**
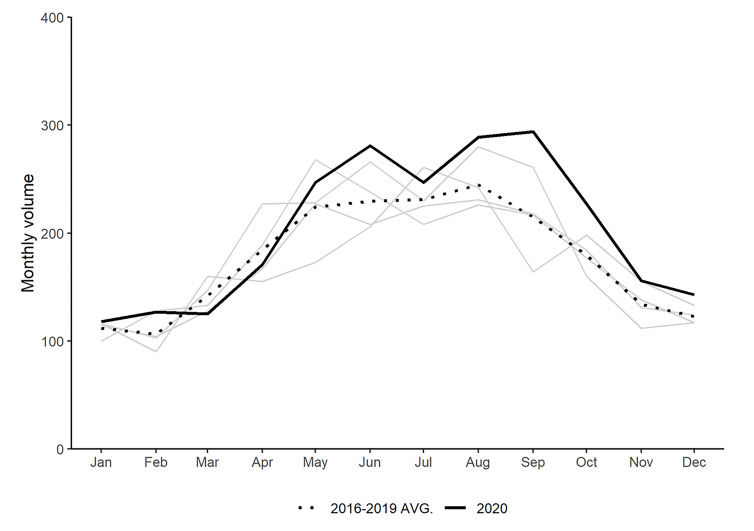
Comparison of monthly pediatric trauma volume by year.

Figure 2 compares the statewide monthly volume of pediatric trauma injuries in 2020 to previous years. March and April 2020 had volumes lower than the historical average, but every other month in 2020 was above the historic trend. In fact, six months in 2020, including every month after July, were the highest monthly volume in any of the past five years. Alternatively, March 2020 saw the lowest volume of any March in the analysis time period.

**Figure 3:**
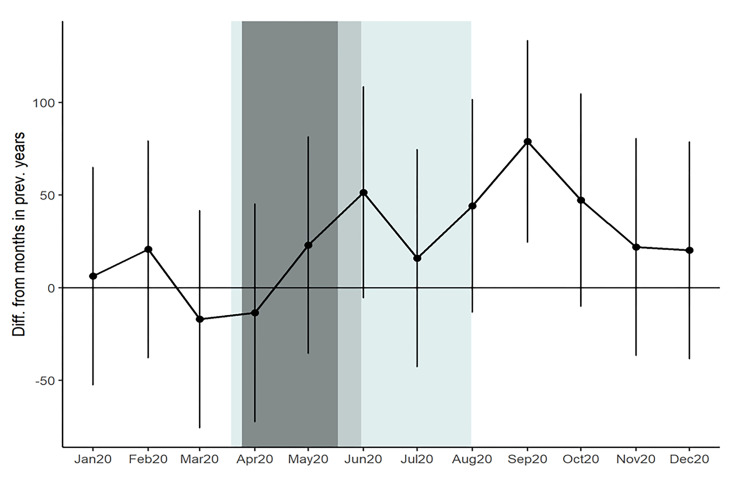
Comparison of 2020 monthly pediatric trauma volume to baseline years.

The descriptive results in Figure 2 are supplemented by statistical comparisons of the months of 2020 to previous years in Table 2. These comparisons show that while March and April 2020 were below the historical average volume, the difference was not statistically significant. The only month of 2020 that differed significantly from the historic trend was September which saw 79 more cases than a typical September from 2016 – 2019. These results are visualized in Figure 3 which plots regression coefficients with 95% confidence intervals and includes shading to denote periods corresponding to Indiana’s SAH order, cancellation of in-person public K-12 schooling, or both. 

**Table 2:** Monthly injury volume for 2020 with comparisons to historic trend.

Month	Total Volume	Mechanism		Location	
		GSW	NAT	Private residence	School
January	118	5	9	69	9
	(+6.25)	(+2.25)	(-3.00)	(+19.00)	(+2.25)
February	127	1	5	63	8
	(+20.75)	(-0.75)	(-7.50)	(+15.50)	(+2.00)
March	125	5	6	74	5
	(-17.00)	(+2.00)	(-11.75**)	(+16.00)	(+0.75)
April	171	2	19	96	0
	(-13.50)	(-1.00)	(+5.50)	(+27.75)	(-9.00**)
May	247	4	17	129	1
	(+23.00)	(+2.50)	(+5.75)	(+55.75**)	(-8.50**)
June	281	6	21	150	0
	(+51.50*)	(+2.25)	(+5.25)	(+65.50**)	(-2.50)
July	247	5	13	116	1
	(+16.00)	(+2.00)	(-4.00)	(+27.50)	(-2.50)
August	289	7	17	133	11
	(+44.25)	(+4.00**)	(-0.50)	(+52.00**)	(-2.25)
September	294	1	18	128	12
	(+79.00***)	(-1.25)	(+4.50)	(+50.50*)	(+0.50)
October	227	4	18	108	6
	(+47.25)	(+0.75)	(+4.50)	(+51.75**)	(-2.50)
November	156	7	10	82	10
	(+22.00)	(+4.75**)	(-4.25)	(+31.00)	(-1.00)
December	143	6	9	96	1
	(+20.25)	(+3.50*)	(-5.00)	(+46.25*)	(-4.75)

Numbers in each cell provide the volume of injuries for a given month in 2020 (row) and type of injury (column). The numbers in parenthesis show the deviation from the historic trend in a given month and injury type. Positive numbers in parenthesis indicate 2020 volume was greater than historic data and negative numbers indicate 2020 volume was below historic trends. Stars indicate statistical significance as follows: * p <0.10, ** p <0.05, *** p <0.01. GSW = gunshot wound, NAT = non-accidental trauma.

**Figure 4:**
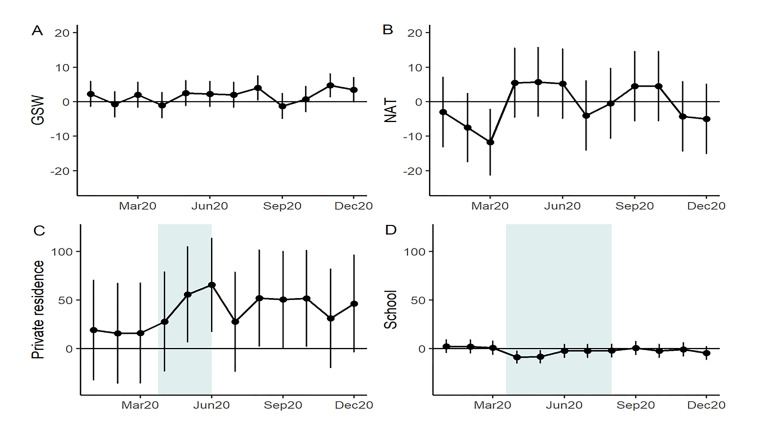
Comparison of 2020 monthly pediatric trauma volume to baseline years by mechanism and location of injury.

Table 2 also highlights two important mechanisms of injury (GSWs and NATs) that have been studied previously in the literature as well as two injury locations (private residences and schools) which were plausibly impacted by SAH orders and cancellation of in-person K-12 public schooling. On a monthly basis, GSWs were comparable to previous years except August and November 2020 which saw a significant elevation from previous years. NATs were significantly lower in March 2020 than previous years (11.75 fewer) but were similar to previous years in all other months. Injuries in private residences were more prevalent in May and June 2020 and injuries in schools were less prevalent in April and May 2020. These periods correspond closely with Indiana’s SAH order and the closure of public K-12 schools. Some school injuries occurred in May and June, despite public school closure, due to students at private schools attending classes in person. Monthly trends are shown for the results above in Figure 4.

## Discussion

Our findings differ from previous estimates of the impact of COVID-19. We find that March and April 2020, the era most impacted by Indiana’s SAH order and in-person public school closures, had lower volumes of pediatric trauma injuries than the recent historical average. However, after these policies were removed, injury volume quickly rose again leading to the second half of 2020 being above the historic trend. In aggregate, this contradicts our hypothesis, based on previous studies, that overall volume in 2020 would decline, but some results here corroborate those earlier analyses.

The reduced volume in March and April 2020, though not significantly different from previous years, aligns with other studies’ results that looked specifically at this time period.^2,5-7,9,14,15^ This reduction corresponds to a time period during which SAH orders were in effect and compliance with these orders was at its greatest.^16^ However, the second half of the year saw some of the highest monthly volumes of any year in the study time period. On an annual basis, this more than offset the reductions in volume seen at the beginning of the pandemic. The net effect was that overall trauma volume for 2020 was greater than previous years. 

This is a surprising result in light of reduced opportunities for pediatric injuries. Throughout 2020, children’s sports leagues were cancelled and less time was spent on playgrounds,^7,17^ more time was spent at home,^16^ and driving was reduced. ^18^ Some of our results offer insights into how these behavior changes manifested in pediatric injury data. We found a significant increase in injuries that took place in private residences, which rose to their peak during Indiana’s SAH order, and a reduction in the proportion of injuries caused by MVCs. There was also a small decrease in the proportion of injuries that took place in outdoor settings or during sporting events.

One possible explanation for the difference in Indiana’s trends from results in other geographies is that Indiana’s policy responses differed. However, the timing of Indiana’s initial policy responses to COVID-19 was largely in line with other states around the country. Indiana’s statewide SAH order lasted from 3/25/20 to 5/18/20.^1^Of the 44 states that implemented some type of SAH order, the median start date was 3/28/20 and the median end date was 5/18/20. The median state’s SAH order lasted 52.5 days, Indiana’s lasted 54. K-12 public schools in Indiana were closed for the remainder of the spring 2020 semester on 3/19/20—the median state closed K-12 schools on 3/17/20.^19^ While this rules out policy mandates as a likely contributor, it does not account for potential differences in enforcement of stated policy. Variation in enforcement of SAH orders and differing attitudes toward social distancing in some areas may have reduced the impact of policies on pediatric trauma volume.

To date, the most comprehensive review of pediatric injury volume during COVID-19 found a 27 percent reduction during 2020.^10^ In that analysis, the overall reduction was driven by decreased volume for minor injuries which offset increases in severe injuries. Reproducing the injury severity categories used in Wells et al. (2022), we found a corresponding reduction in the lowest severity injuries, but an increase in “mild” injuries (the largest group) and no change in more severe injury categories compared to baseline years (analysis not shown). The difference in results may be due to differences in samples: our sample had considerably more white children (80% vs. 43%) and excluded children over age 15.

In our analysis, injuries did not increase proportionately across all mechanisms in 2020: falls, MVCs, strikes by objects, and NAT all became less common as pedestrians strikes by vehicles, GSWs, and “other” mechanisms became more common. Concerningly, the volume of GSWs in 2020 grew by over 50% from baseline years. These injuries were split among accidental/undetermined and intentional (assault) injuries, but corroborate trends of increased violent injuries reported in other studies.^11,20-22^ Increased gun violence could be attributed to increased substance abuse during the pandemic and social isolation induced by policies such as SAH orders.^22^ This troubling trend warrants additional research.

Another mechanism of interest in the literature, NATs, fell slightly during 2020 compared to 2016-2019. This reduction occurred primarily in March 2020 during the initial response to COVID-19. It is unlikely that this indicates a true change in the prevalence of child maltreatment, but rather it reflects a loss of detection of NATs as children spent less time in schools and more time at home away from mandatory reporters who typically report these types of injuries.^23-25^

The limitations of this study stem from the use of a large, statewide trauma registry. While these data provide a longer timeframe and more generalizable sample of cases than some previous work, the tradeoff is less detail on individual cases. Dates were aggregated to months making short-term analyses focused on the early weeks of the pandemic impossible. In some cases, location data were available at the county level, but analysis was carried out at the state level due to missing county information. Future studies could utilize large datasets that retain granularity of injury timing and location and incorporate measures of adherence to COIVD-19 policy responses. This would better identify the effects of SAH policies and cancellation of in-person schooling on injury patterns. 

In summary, this analysis leverages a statewide trauma database to provide a more generalizable and longer-term look at the response of pediatric trauma volume to COVID-19. This analysis contributes to the growing literature on changes in patterns of injury and treatment in the wake of COVID-19, corroborating some results but showing that volume increased overall during 2020. As policymakers and hospital administrators allocate resources in response to the ongoing COVID-19 pandemic, data like these emphasize that the need to manage traumatic injuries remains high. 

**Acknowledgements**

The authors would like to acknowledge contributions from staff in the Division of Trauma and Injury Prevention at the Indiana Department of Health. Specifically, Ramzi Nimry who provided an overview of the trauma patient registry and outlined the data request process, Trinh Dinh who helped develop and prepare the data request, and Timothy Miller who provided technical assistance with the data request.

1. The statewide SAH mandate was in effect for all counties from 3/25/20 to 5/1/20 with several counties re-opening on 5/1/20, several more on 5/4/20, and all but one county reopening on 5/18/20. Marion County, the state’s most populous county, reopened on 5/31/20.
